# STONE Score as a Triage Tool to Guide Computed Tomography of the Kidneys, Ureters, and Bladder (CT-KUB) Requests in Suspected Renal Colic: A Quality Improvement Initiative

**DOI:** 10.7759/cureus.92080

**Published:** 2025-09-11

**Authors:** Omar Nasr Hassouba, Abdulaziz Alsamani Abdullah Omar, Manahil Awan, Shahzad Ahmad, Mawada Taha, Sharmila Venkatachalapathi, Mohamed K Abouelsadat, Albina Mercy, Abdelrahman Sahnon Abaker Sahnon, Usama Shafique, Dodi I Herman

**Affiliations:** 1 Emergency, Shrewsbury and Telford Hospital NHS Trust, Shrewsbury, GBR; 2 General Surgery, Prince Othman Digna Teaching Hospital, Port Sudan, SDN; 3 Executive and Special Ward, Liaquat National Hospital, Karachi, PAK; 4 Surgery, Liaquat National Hospital, Karachi, PAK; 5 General Surgery, The National Ribat University, Khartoum, SDN; 6 Internal Medicine, Periyar Government Hospital, Mayiladuthurai, IND; 7 Internal Medicine, Vadamalayan Hospitals [P] Ltd., Dindigul, IND; 8 Vascular Surgery, Royal Free Hospital, London, GBR; 9 Internal Medicine, Davao Medical School Foundation, Inc., Davao, PHL; 10 General Practice, Medical Council of Ireland, Dublin, IRL; 11 General Surgery, Royal College of Surgeons of Edinburgh, Edinburgh, GBR; 12 General Surgery, Azra Naheed Medical College, Lahore, PAK

**Keywords:** ct-kub, diagnostic accuracy, emergency medicine, renal colic, stone score, ureteric stone

## Abstract

Introduction

Urolithiasis is a frequent cause of emergency department (ED) visits, with computed tomography (CT) being the gold standard for diagnosis. Excessive imaging increases radiation exposure and healthcare costs. The STONE score is a validated clinical prediction tool, designed to estimate the probability of ureteric stones and reduce unnecessary imaging.

Objective

The main objective of this study is to evaluate the diagnostic accuracy of the STONE score in patients presenting with flank pain.

Methodology

This is a cross-sectional retrospective review conducted at the Shrewsbury and Telford Hospital NHS Trust (SATH), Shrewsbury, England, over a four-month period from April 1, 2023, to July 31, 2023. This quality improvement initiative reviewed 81 eligible ED patients who underwent computed tomography of the kidneys, ureters, and bladder (CT-KUB) for suspected ureteric stones. Demographic, clinical, laboratory, and imaging data were collected. STONE scores were calculated for all patients. Diagnostic performance was assessed using receiver operating characteristic (ROC) curve analysis.

Results

The mean age was 38.5 ± 16.1 years; 35 (43.2%) were male. Ureteric stones were confirmed in 15/19 (78.9%) high-risk, 9/45 (20%) moderate-risk, and 0/17 (0%) low-risk patients. The STONE score yielded an area under the curve (AUC) of 0.879, with a sensitivity of 91.7% and a specificity of 66.7%. Alternative diagnoses included gallbladder stones, appendicitis, cystitis, diverticulitis, hydronephrosis, renal angiomyolipoma, polycystic kidney disease (PCKD), pyelonephritis, and small bowel obstruction (SBO).

Conclusion

The STONE score demonstrates good diagnostic accuracy, particularly in high-risk patients, and may help reduce unnecessary CT imaging and radiation exposure in the ED.

## Introduction

Flank pain is a common and clinically significant presentation in the emergency department (ED), frequently attributable to renal or ureteric pathology. Urolithiasis, in particular, is a major contributor to this symptom burden, with a lifetime prevalence estimated at approximately 5% in the general population and up to 8% of such ED visits resulting in hospital admission [1]. The diagnostic evaluation of suspected renal colic requires a systematic approach that integrates a detailed clinical history, focused physical examination, targeted laboratory investigations, and judicious use of imaging modalities.

Computed tomography of the kidneys, ureters, and bladder (CT-KUB) is considered the diagnostic gold standard for urolithiasis, due to its high sensitivity and specificity across stone compositions and anatomical locations. CT is especially indicated in patients with high-risk clinical profiles, including a prior history of nephrolithiasis, underlying renal disease, known malignancy, and features suggestive of obstructive uropathy with infection (such as fever, leukocyturia, and elevated white cell count), or previous urological interventions, such as lithotripsy or ureteral stent placement [2].

Nevertheless, a substantial proportion of urinary stones, particularly those <5 mm in diameter, are uncomplicated and pass spontaneously without intervention, and there is no strong evidence that routine CT in such cases alters long-term clinical outcomes [3,4]. From a pathophysiological standpoint, recurrent stone formation is especially prevalent among younger adults due to underlying metabolic abnormalities (e.g., hypercalciuria, hyperoxaluria, hypocitraturia), dietary factors, and genetic predispositions [5]. Consequently, recurrent exposure to CT imaging in this demographic raises cumulative ionizing radiation doses, which have been associated with a small but significant increase in lifetime malignancy risk. In response to these concerns, multiple clinical risk prediction tools have been developed to estimate the probability of ureteric stones, without immediate reliance on imaging.

Such scoring systems, among which the STONE score is one of the most widely validated, use combinations of demographic variables, symptom duration, associated gastrointestinal symptoms, and urinalysis findings to stratify patients into low-, moderate-, and high-risk categories for urolithiasis [6]. Beyond diagnostic utility, these tools have demonstrated the potential to reduce healthcare costs, decrease patient radiation exposure, and optimize ED workflow by prioritizing imaging for those most likely to benefit from immediate diagnosis. Moreover, the overuse of CT not only contributes to avoidable radiation exposure but also increases ED length of stay, consumes radiology resources, and delays definitive patient management. A targeted, evidence-based triage approach leveraging objective scoring systems will offer an opportunity to improve diagnostic efficiency, promote patient safety, and align with modern quality improvement initiatives aimed at high-value care.

## Materials and methods

Study setting and context

This cross-sectional retrospective review was conducted at the Shrewsbury and Telford Hospital NHS Trust (SATH), Shrewsbury, England, over a four-month period from April 1, 2023, to July 31, 2023.

Methods

The cross-sectional retrospective review's data were retrieved by the clinical audit team from the hospital information system and patient records, identifying all individuals who had presented to the Accident & Emergency (A&E) Department with acute flank pain and subsequently underwent CT-KUB during the study period. For each patient, demographic data (age and sex), relevant medical history (including pain onset and duration, presence of nausea or vomiting), and laboratory results (urinalysis, renal function tests, and infection markers such as neutrophil count and white blood cell count) were recorded. Hematuria was defined as the presence of red blood cells on urine dipstick or microscopy, while leukocytes on urinalysis were taken as indicative of urinary tract infection (UTI). Exclusion criteria included pregnancy, age under 18 years, trauma-related flank pain, confirmed UTI, inability to communicate (including mutism or reduced consciousness), known malignancy, and unstable vital signs at presentation. CT-KUB reports, issued by consultant radiologists, served as the diagnostic gold standard for ureteric stones. The STONE score for each patient was calculated according to its five parameters, including sex, pain duration, race, gastrointestinal symptoms, and hematuria status, and stratified into low-risk (0-5 points), moderate-risk (6-9 points), and high-risk (10-13 points) categories, as outlined in Table 1. Patient stratification according to the STONE score. Scoring system adapted from Safaei et al. [6].

**Table 1:** Parameters, criteria, points, and evaluation of STONE scoring system Evaluation: 0-5 points: low risk; 6-9 points: moderate risk; 10-13 points: high risk. Patient stratification according to the STONE score. Scoring system adapted from Safaei et al. [6].

Parameters	Criteria	STONE scoring system
Sex	Male	2
	Female	0
Duration of the pain upon admission	>24 hours	0
	6-24 hours	1
	<6 hours	3
Race	Non-caucasian	0
	Caucasian	3
Nausea and vomiting	None	0
	Nausea alone	1
	Nausea plus vomiting	2
Hematuria on urine dipstick	Absent	0
	Present	3

Statistical analysis

Data analysis was performed using IBM SPSS Statistics for Windows, Version 24 (Released 2016; IBM Corp., Armonk, NY, USA) [7]. Categorical (qualitative) variables were summarized as frequencies and percentages, while continuous (quantitative) variables were presented as mean values with standard deviations. The Shapiro-Wilk test was applied to assess the normality of distribution for continuous variables. Comparative analyses of categorical variables were conducted using the Chi-square test or Fisher’s exact test, as appropriate, based on expected cell counts. Diagnostic accuracy of the STONE score was evaluated using receiver operating characteristic (ROC) curve analysis, with the area under the curve (AUC) calculated to quantify overall performance. Sensitivity, specificity, positive predictive value (PPV), and negative predictive value (NPV) were also determined. The optimal score threshold for stratifying patients into high- and low-risk categories was identified from the ROC-derived cut-off value. A two-tailed p-value of less than 0.05 was considered statistically significant.

Ethical considerations

The Clinical Audit Department at the Shrewsbury and Telford Hospital NHS Trust reviewed the study and concluded that it does not qualify as human research.

## Results

**Figure 1:**
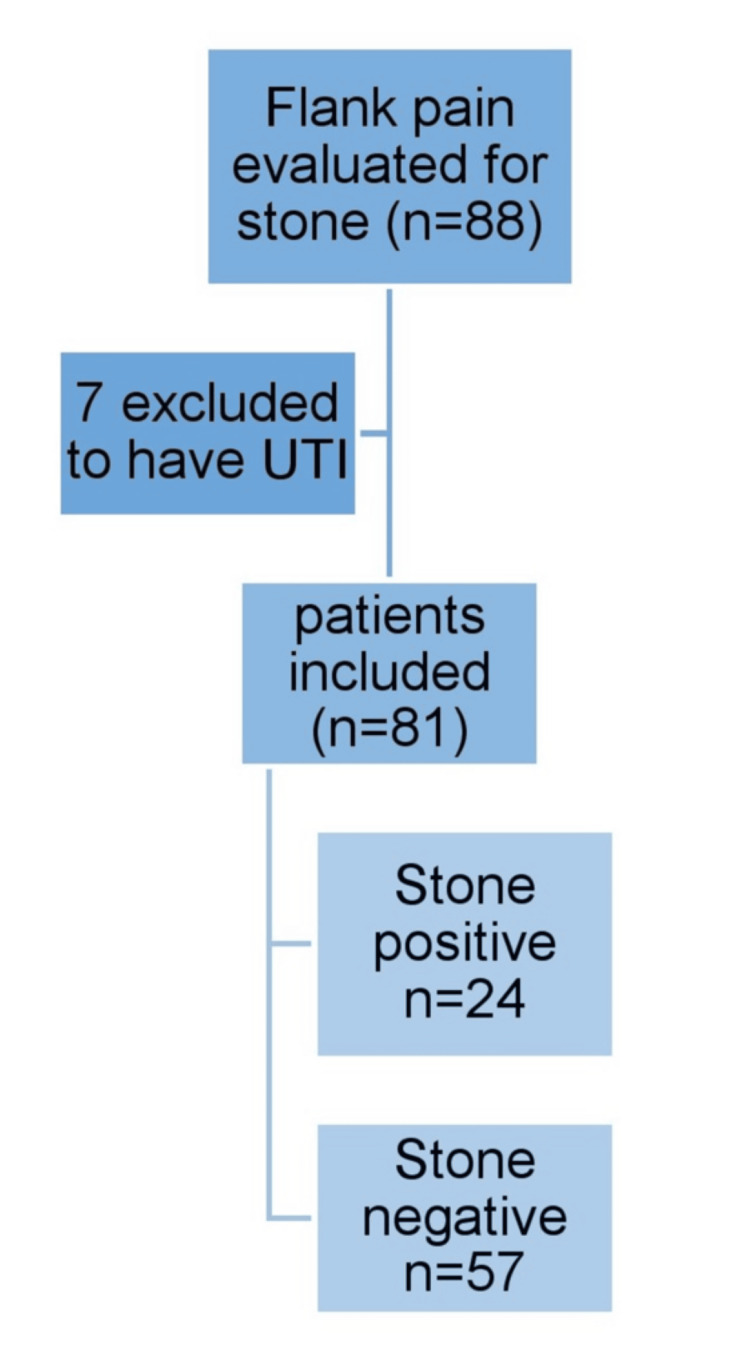
Flow chart of the study UTI: urinary tract infection

Reviews were conducted on 88 patients in total, but only 81 were enrolled in the study after meeting the requirements for inclusion. Figure 1 represents the flowchart illustrating the patient selection process and diagnostic outcomes in a study evaluating individuals presenting with flank pain for suspected ureteric stones. Initially, a total of 88 patients were assessed. Of these, seven patients were excluded from the study due to a confirmed diagnosis of UTI, which could mimic similar symptoms but does not align with the study’s focus on urolithiasis. After these exclusions, 81 patients remained eligible and were included in the final analysis. Among these, 24 patients were diagnosed as stone-positive, indicating the presence of ureteric stones, while the remaining 57 patients were classified as stone-negative, having no radiological or clinical evidence of stones. This stepwise inclusion and exclusion process highlights the study’s methodological rigor in defining a relevant patient cohort for evaluating the diagnostic accuracy of stone detection tools.

Table 2 demonstrates the patients assessed for ureteric stones. Several factors were significantly associated with stone positivity. Male patients were more likely to have stones (62.5% vs. 35.1%, p < 0.001), as were those presenting with hematuria (87.5% vs. 56.1%, p = 0.003) and gastrointestinal symptoms such as nausea and vomiting (p = 0.046). A shorter duration of pain, particularly less than six hours, was also strongly linked to stone presence (41.7% vs. 1.7%, p < 0.001). While age and race showed no statistically significant differences, trends suggested more cases among older individuals. Overall, male gender, early symptom onset, hematuria, and nausea were key indicators of ureteric stones.

**Table 2:** Comaprsion of STONE criteria among patients according to presence or absence of stone ^a^Chi-square test; ^b^Fisher exact test; *Statistically significant as p < 0.05. There was no timing for three patients.

Parameter	Total n (%)	Stone positive n (%)	Stone negative n (%)	p^a^
Gender				
Female	46 (66.8)	9 (37.5)	37 (64.9)	<0.001*^a^
Male	35 (43.2)	15 (62.5)	20 (35.1)	
Age				
<25 years	5 (39.5)	0 (41.7)	5 (8.8)	0.099^a^
25-39 years	17 (60.5)	3 (12.5)	14 (24.5)	
40-59 years	27	11 (45.8)	16 (28.1)	
>60 years	32	10 (41.7)	22 (38.6)	
Race				
Caucasian	76 (93.8)	23 (95.8)	53 (93)	0.725^b^
Non-caucasian	5 (6.2)	1 (4.2)	4 (7)	
Nausea and vomiting history				
Nausea	29 (35.8)	13 (54.2)	16 (28.1)	0.046*^a^
Both	7 (9.9)	3 (12.5)	5 (8.7)	
No	44 (54.3)	8 (33.3)	36 (63.2)	
Duration of the pain				
<6 h	11 (13.6)	10 (41.7)	1 (1.7)	<0.001*^a^
6 h to 1 day	12 (14.8)	6 (25)	6 (10.5)	
>1 day	55 (76.9)	8 (33.3)	47 (82.5)	
Hematuria				
Yes	53 (56.4)	21 (87.5)	32 (56.1)	0.003*^a^
No	28 (44.6)	3 (12.5)	25 (43.9)	

**Figure 2:**
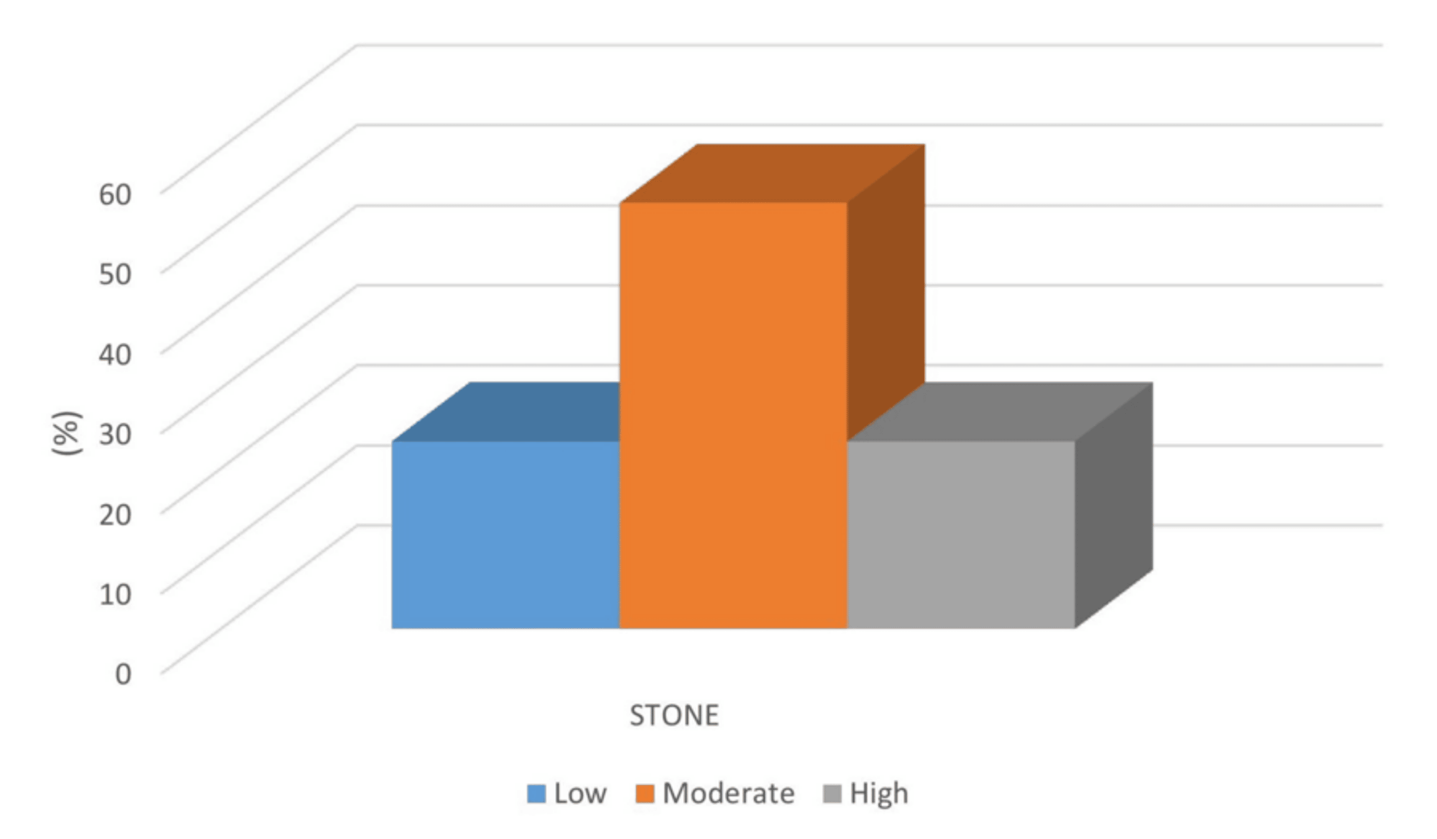
Prevalence of ureteral stones according to the STONE score

Based on the STONE scoring system, 17 patients (20.9%) were classified as low-risk, and none of them were diagnosed with ureteric stones. In the high-risk group, which included 19 patients (23.4%), 15 were confirmed to have stones. Among the 45 patients (55.7%) in the moderate-risk group, nine were stone-positive. These findings are illustrated in Figure 2.

**Figure 3:**
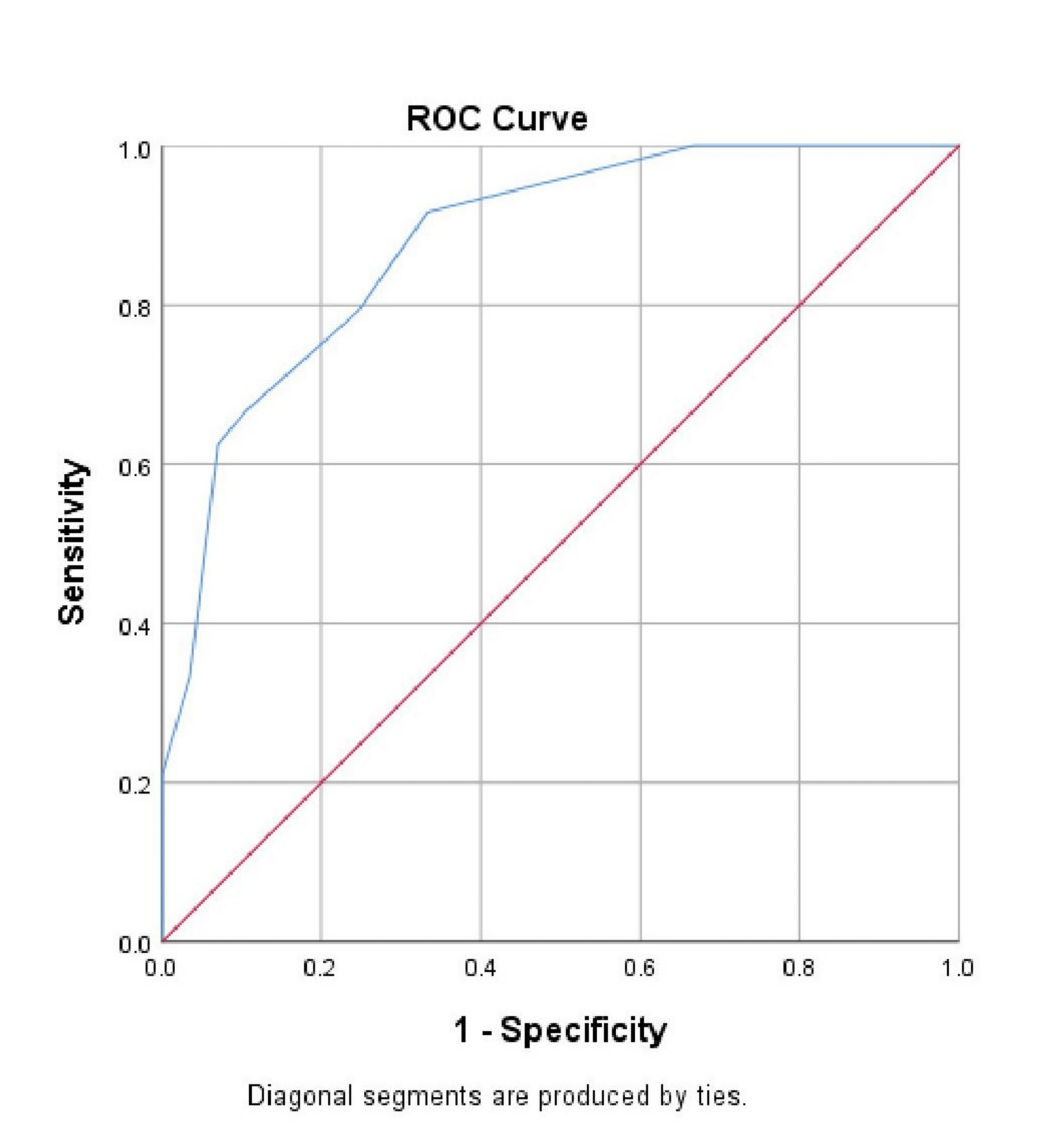
Receiver operating characteristics (ROC) of the STONE scoring system

Figure 3 shows the ROC curve, and the diagnostic performance of the STONE scoring system is summarized in Table 3, demonstrating strong overall accuracy in predicting ureteric stones. At a cutoff value of 6.5, the STONE score achieved an AUC of 0.879, indicating excellent discriminative ability. The sensitivity was notably high at 91.7%, meaning the scoring system correctly identified most patients who had ureteric stones. The specificity was 66.7%, reflecting a moderate ability to correctly identify those without stones. Furthermore, the PPV was 73.4%, indicating that approximately three out of four patients classified as high-risk truly had stones. The NPV was even higher at 88.9%, suggesting that the score is particularly effective at ruling out stones in low-risk patients. These findings underscore the utility of the STONE score as a reliable diagnostic tool, especially in emergency settings, where quick and accurate triage is essential.

**Table 3:** Diagnostic performance of STONE score NPV: negative predictive value; PPV: positive predictive value; AUC: area under the curve

	Cut off	AUC	Sensitivity	Specificity	PPV	NPV
STONE	6.5	0.879	91.7	66.7	73.4	88.9

Types and frequency of alternative diagnoses by the STONE score are shown in Table 4. Patients with various abdominal and renal diagnoses were assigned STONE scores that classified them into low-, moderate-, or high-risk categories for ureteric stones. For example, appendicitis cases had scores of 3 (low risk) and 6 (moderate risk), while cystitis patients scored 6 and 7, both within the moderate-risk range. Diverticulitis cases consistently scored 6, also placing them in the moderate-risk group. Hydronephrosis showed greater variability, with scores ranging from 3 (low risk) to 10 (high risk). A patient with left renal angiomyolipoma was assigned a score of 8, corresponding to moderate risk, and polycystic kidney disease (PCKD) cases scored 6, also moderate risk. Pyelonephritis with fluid presented scores from 4 and 5 (low risk) to 6 (moderate risk). Small bowel obstruction (SBO) cases were scored 3, indicating low risk. This range of scores illustrates how the STONE system stratifies different diagnoses based on their likelihood of ureteric stones.

**Table 4:** Types and frequency of alternative diagnoses by the STONE score PCKD: polycystic kidney disease; SBO: small bowel obstruction; Lt: left

Diagnosis	Scores (Risk)
Appendicitis	3 (Low), 6 (Moderate)
Cystitis	6 (Moderate), 7 (Moderate)
Diverticulitis	6 (Moderate), 6 (Moderate), 6 (Moderate)
Hydronephrosis	3 (Low), 10 (High)
Lt. renal angiomyolipoma	8 (Moderate)
PCKD	6 (Moderate)
Pyelonephritis fluid	4 (Low), 5 (Low), 6 (Moderate)
SBO	3 (Low)

## Discussion

The present study provides further evidence supporting the validity of the STONE scoring system as a diagnostic tool for ureteric stones in the emergency setting. While clinical evaluation, comprising detailed history taking and physical examination, remains the cornerstone of initial assessment, structured scoring systems such as STONE offer an objective and reproducible approach to risk stratification. These tools can be particularly beneficial in resource-limited environments or where diagnostic imaging may be delayed. In the context of diagnostic testing, when the pre-test probability of disease is low, a test with high sensitivity is preferred to rule out the condition effectively.

The STONE score demonstrated a high sensitivity of 91.7% and a specificity of 66.7% in our study, yielding an AUC of 0.879. This performance suggests that the STONE score is particularly well-suited as a rule-out tool in patients with suspected ureteric stones. However, the relatively modest specificity may lead to false positives, which underscores the need for potential refinement of the scoring algorithm or the development of alternative models better tailored to specific populations. Türk and Ün [8], in a retrospective analysis of a Turkish cohort, identified male sex, hematuria, a family history of urolithiasis, and gastrointestinal symptoms such as nausea and vomiting as independent predictors of ureteral stones. Notably, these variables are also incorporated into the components of the STONE score, reinforcing its construct validity. Our findings are consistent with their observations, suggesting that the STONE score captures key clinical features associated with urolithiasis across different populations [8]. Comparative studies have reported variable diagnostic performance for the STONE score. For instance, a large-scale study involving 1,013 patients found that 856 were ultimately diagnosed with urinary stones, demonstrating a disease prevalence of approximately 84.5% in their cohort.

In that study, the AUC for the STONE score was reported at 0.71, with an optimal cutoff of 9 yielding a sensitivity of 87.9% and a specificity of 45.9% [9]. These values indicate that, although the score is reasonably effective in identifying true positives, its lower specificity may result in overdiagnosis or unnecessary imaging. In an external validation study by Hernandez et al., a notable discrepancy was observed in the prevalence of ureteric stones among low-risk patients, with 24.1% testing positive - significantly higher than the 8.3%-9.2% reported in the original STONE cohort. Their findings highlight the necessity of re-evaluating the scoring thresholds, particularly for low- and moderate-risk groups, where the misclassification rate may be clinically consequential [10].

Cochon et al. further explored the role of imaging by comparing the performance of the STONE score with that of CT. Their findings suggested that CT did not provide additional diagnostic value in high-risk patients when the STONE score was applied, supporting the use of the scoring system as a frontline triage tool [11]. Additionally, while the inclusion of hydronephrosis as an ultrasonographic marker showed marginal improvement in predictive performance among low- and moderate-risk patients, it did not enhance diagnostic accuracy in the high-risk group [12]. The integration of point-of-care ultrasound (US) into scoring systems remains an area of active investigation. In particular, the STONE PLUS scoring model, which incorporates US findings, did not demonstrate significant performance gains over the original STONE score in a recent evaluation, suggesting limited added value in certain clinical scenarios [13].

The age distribution of patients may also impact the performance of the STONE score. A validation study by Schoenfeld et al. demonstrated the tool's reliability in a relatively young cohort, with a mean age of 37 years [14]. Similarly, our study, with a mean participant age of 38 years, found the score to be particularly effective in the high-risk group. However, one notable limitation was the “race” variable, which was uniformly negative in our cohort. The absence of racial variability may have contributed to diminished discriminative capacity in the low- and moderate-risk strata, suggesting that the role of this variable should be reassessed, especially in ethnically homogeneous populations.

## Conclusions

This study supports the STONE score as a valid and practical tool for diagnosing ureteric stones, particularly in high-risk patients. With a sensitivity of 91.7% and an AUC of 0.879, it performs well as a rule-out test and can aid in reducing unnecessary CT imaging. Its objective structure complements clinical evaluation and is especially useful in resource-limited or high-volume emergency settings. However, moderate specificity, and the limited applicability of certain variables - such as race - suggest a need for refinement in specific populations. Overall, the STONE score remains a valuable adjunct in the diagnostic pathway for suspected urolithiasis.
